# Case Report: Primary malignant melanoma of the uterine cervix with early recurrence following radical surgery and immunotherapy

**DOI:** 10.3389/fonc.2026.1970684

**Published:** 2026-09-21

**Authors:** Alexandru-George Filipescu, Ana-Maria Cioca, Alex Gabriel Strajean, Andreea Lungu, Andreea Boiangiu

**Affiliations:** 1 Department of Obstetrics and Gynecology, Elias University Emergency Hospital, Bucharest, Romania; 2 Department of Obstetrics and Gynecology, Carol Davila University of Medicine and Pharmacy, Bucharest, Romania

**Keywords:** case report, immune checkpoint inhibitor, malignant melanoma, mucosal melanoma, pembrolizumab, primary cervical melanoma, uterine cervix

## Abstract

**Background:**

Primary malignant melanoma of the uterine cervix (PMMC) is an exceptionally rare mucosal malignancy with an aggressive clinical behavior and no established standard of care. Distinguishing a primary cervical melanoma from metastatic disease is particularly challenging and requires integration of pathological findings with comprehensive evaluation for an alternative primary site.

**Case presentation:**

We report a 67-year-old postmenopausal woman presenting with vaginal bleeding and an apparently benign endocervical polyp. Histopathological and immunohistochemical examination established the diagnosis of malignant melanoma, with diffuse SOX10, melanoma cocktail, and PRAME positivity and a Ki-67 proliferation index of approximately 40–45%. Molecular testing showed no detectable BRAF mutation. Dermatological assessment and systemic imaging identified no alternative primary melanoma or evidence of distant metastatic disease at initial staging. The patient underwent radical hysterectomy with bilateral pelvic lymphadenectomy and para-aortic lymphadenectomy and omental biopsy. Pelvic lymphadenectomy included bilateral external iliac nodal dissection, with no metastatic involvement identified in the examined lymph nodes. Postoperative pembrolizumab was subsequently initiated. Despite apparently localized disease and multimodal treatment, biopsy-confirmed vaginal recurrence developed approximately two months after surgery. Restaging ^18^F-FDG PET/CT demonstrated metabolically active disease involving the vaginal cuff and vagina, together with findings suspicious for external iliac nodal and hepatic involvement. Palliative external-beam radiotherapy was subsequently administered for hemostatic and locoregional control.

**Conclusion:**

This case highlights the aggressive and unpredictable course of PMMC, with early histologically confirmed recurrence following radical surgery and postoperative immune checkpoint inhibition. The rapid progression observed in our patient, considered alongside the heterogeneous responses to PD-1 blockade reported in the literature, underscores the therapeutic uncertainty surrounding this rare malignancy and the need for improved predictors of treatment response.

## Introduction

Primary malignant melanoma of the uterine cervix (PMMC) represents an extremely uncommon form of mucosal melanoma involving the female genital tract. Although melanoma most often develops in the skin, primary tumors may also arise from melanocytes located at mucosal sites. Mucosal melanomas account for only a small proportion of all melanomas, and involvement of the female genital tract represents an especially uncommon subset (3). Genital tract involvement is uncommon and, when present, predominantly affects the vulva or vagina. Cervical origin is particularly unusual, with relatively few cases described in the literature (1, 2).

Cervical melanoma typically presents with nonspecific gynecological symptoms, with abnormal vaginal bleeding being the most frequently reported clinical manifestation (1, 8). Histopathological diagnosis may be challenging, particularly in poorly differentiated or amelanotic tumors, which can morphologically resemble other high-grade malignancies. Immunohistochemical evaluation is therefore essential to confirm melanocytic differentiation and support an accurate diagnosis (8). Once melanoma of the cervix is identified, establishing whether the tumor is primary or represents metastatic involvement is a critical step in the diagnostic work-up. This distinction requires the exclusion of a cutaneous, ocular, or other mucosal primary melanoma through comprehensive clinical and systemic evaluation (1, 2).

Due to the exceptional rarity of PMMC, evidence regarding optimal management remains limited and standardized therapeutic strategies have not been established. Surgery represents the mainstay of treatment for apparently localized disease (1, 2), whereas the roles of radiotherapy and systemic treatment, including immune checkpoint inhibition, remain less clearly defined (1–4). The aggressive biological behavior of the disease and the limited available evidence make treatment decisions particularly challenging.

We report a rare case of primary cervical melanoma in a 67-year-old woman who developed early biopsy-confirmed vaginal recurrence following radical surgery and subsequent pembrolizumab therapy. The exceptionally early, biopsy-confirmed recurrence following apparently complete surgical treatment and postoperative PD-1 blockade represents a notable feature of this case, underscoring the aggressive behavior of PMMC and the need for reliable predictors of immunotherapy response. This case, together with a focused literature review, highlights the diagnostic complexity, aggressive clinical behavior, and therapeutic challenges of this uncommon malignancy.

## Methods

This case report was prepared in accordance with the CARE guidelines. Clinical data were retrospectively collected from the patient’s medical records, including clinical presentation, imaging findings, histopathological and immunohistochemical results, treatment, and available follow-up data. All patient-related information was anonymized. Written informed consent for publication of the clinical data and images was obtained from the patient.

A focused literature review was performed to identify previously reported cases and relevant studies on primary malignant melanoma of the uterine cervix. PubMed/MEDLINE was searched using combinations of the terms “primary cervical melanoma, “ “malignant melanoma of the uterine cervix, “ “mucosal melanoma, “ “pembrolizumab, “ and “immune checkpoint inhibitors”. The literature search focused on publications addressing diagnostic criteria, clinicopathological characteristics, treatment strategies, recurrence patterns, prognosis, and the use of immune checkpoint inhibitors. Reference lists of relevant publications were additionally screened to identify potentially eligible studies. The literature search was updated prior to manuscript submission.

## Case report

A 67-year-old postmenopausal woman presented to our hospital with vaginal bleeding persisting for two weeks. Her gynecological history included two full-term vaginal deliveries, with no history of miscarriage or abortion. Natural menopause had occurred at the age of 47. Her medical history was otherwise unremarkable, with no previous diagnosis of malignancy. Two years before the current presentation, she had undergone abdominal and pelvic computed tomography (CT) for an unrelated indication, which revealed no abnormalities of the uterus, cervix, or regional lymph nodes. Multiple hypodense lesions measuring up to 29.7 mm were identified in both hepatic lobes and had initially been interpreted radiologically as benign hepatic lesions, including possible adenomas, based on their imaging appearance and subsequent stability over time. At admission, pelvic examination revealed an endocervical polyp protruding through the cervical os. The lesion was excised and submitted for histopathological examination. Microscopic evaluation revealed fibrinohemorrhagic material containing highly pleomorphic cells with marked variation in size and shape, prominent nuclear atypia, enlarged hyperchromatic nuclei, and a markedly increased nuclear-to-cytoplasmic ratio. These findings raised suspicion of a high-grade malignant neoplasm.

To further characterize the tumor, an extensive immunohistochemical (IHC) panel was performed. The neoplastic cells showed strong and diffuse positivity for SOX10 and a melanoma cocktail, consistent with melanocytic differentiation. The complete immunohistochemical profile and its contribution to the differential diagnosis are summarized in **Table 1**. Epithelial markers, including AE1/AE3, CK8/18, CK5/6, and CK7, were negative in the tumor cells, with appropriate internal positive staining in the residual squamous epithelium. Neuroendocrine markers, including synaptophysin and chromogranin, as well as the lymphoid markers CD20 and CD3, were negative. PAX8, p16, and p63 were also negative in the tumor cells, arguing against Müllerian and squamous epithelial differentiation.

**Table 1:** Immunohistochemical profile and differential diagnosis of the cervical tumor.

Marker	Result	Diagnostic relevance
SOX10	Strong, diffuse positive	Supports melanocytic differentiation
Melanoma cocktail	Strong, diffuse positive	Supports melanocytic differentiation
PRAME	Strong positive	Supports malignant melanocytic neoplasm
AE1/AE3	Negative	Argues against epithelial malignancy
CK8/18, CK5/6, CK7	Negative	Argues against epithelial differentiation
PAX8	Negative	Argues against Müllerian epithelial origin
p63	Negative	Argues against squamous differentiation
p16	Negative	Does not support HPV-associated cervical carcinoma
Synaptophysin, chromogranin	Negative	Argues against neuroendocrine neoplasm
CD20, CD3	Negative	Argues against B- or T-cell lymphoid neoplasm
Ki-67	~40–45%	High proliferative activity

Histologically, the tumor showed an infiltrative growth pattern composed of solid nests, sheets, and anastomosing trabeculae separated by fibrovascular septa. The neoplastic cells had scant cytoplasm, irregular nuclear contours, vesicular chromatin, and prominent eosinophilic nucleoli, with numerous mitotic figures. PRAME showed strong positivity, further supporting the diagnosis of malignant melanoma, while Ki-67 immunostaining revealed a high proliferation index of approximately 40–45%. Fragments of non-keratinized squamous epithelium consistent with exocervical mucosa were identified at the surface of the tumor, confirming the cervical origin of the biopsy specimen. Additional pathological parameters, including precise tumor thickness, depth of stromal invasion, ulceration status, lymphovascular invasion, and a standardized mitotic rate, were not available from the original pathological report.

The morphological and immunohistochemical findings were consistent with malignant melanoma involving the uterine cervix. Additional molecular profiling for NRAS or KIT alterations was not available, and PD-L1 expression was not assessed. Given the rarity of primary cervical melanoma and the need to exclude metastatic disease, further imaging was performed. Abdominopelvic magnetic resonance imaging (MRI) revealed a mildly enlarged uterus measuring 7 × 2.3 × 5 cm and a myometrial nodular lesion consistent with a leiomyoma. Three hypointense foci at the external cervical os were interpreted as post-polypectomy changes. No ascites or pelvic lymphadenopathy was identified, although several subcentimeter bilateral inguinal and external iliac lymph nodes were noted. The previously identified hepatic lesions remained stable, without radiological evidence of progression. No suspicious abnormalities were identified in the visualized bony structures.

Following completion of the diagnostic work-up and multidisciplinary evaluation, radical surgical treatment was undertaken. The patient underwent radical hysterectomy with bilateral pelvic and para-aortic lymphadenectomy and omental biopsy. All surgical specimens were submitted for histopathological examination to assess tumor extent, lymph node involvement, and resection margins.

To further exclude an occult cutaneous primary melanoma, a comprehensive dermatological examination was performed. Three clinically suspicious pigmented lesions were identified at different anatomical sites (**Figure 1**). Punch biopsies were obtained and submitted for histopathological examination.

**Figure 1:**
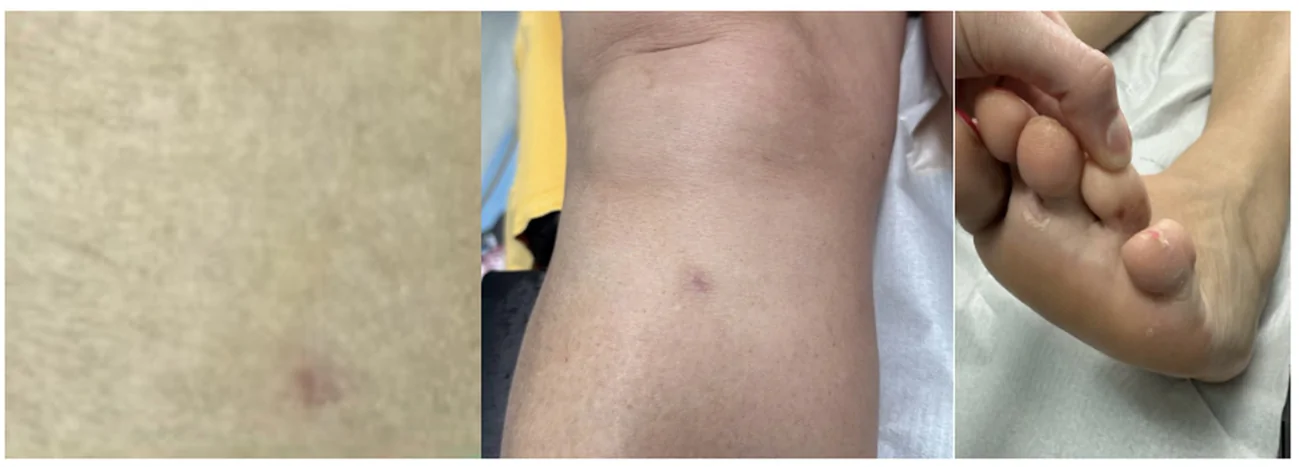
Three clinically suspicious pigmented skin lesions selected for biopsy, shown from left to right: left anterior tibia; right anterior tibia; and lateral aspect of the fourth toe of the left foot. All three lesions were histologically benign.

Histopathological examination of the skin biopsies revealed no evidence of melanocytic proliferation. The two tibial lesions showed no significant pathological abnormalities, while the lesion on the fourth toe was diagnosed as subacute spongiotic dermatitis consistent with eczema.

Microscopic examination of the surgical specimens showed no involvement of the vaginal wall. Focal ulcerative cervicitis, mature and immature squamous metaplasia at the squamocolumnar junction, and moderate ectocervical dysplasia were identified. No malignant involvement of the omentum or examined pelvic and para-aortic lymph nodes was detected. Additional immunohistochemical evaluation of the surgical specimens revealed no residual melanoma and the vaginal resection margin was free of malignant involvement.

Further staging with cerebral and thoracic computed tomography (CT) showed no evidence of distant metastatic disease. No dedicated ophthalmologic or otorhinolaryngologic evaluation for an occult ocular or sinonasal mucosal primary was documented. However, no alternative primary melanoma was identified on systemic imaging, including the subsequent whole-body ^18^F-FDG PET/CT. Taken together with the negative dermatological work-up and the cervical localization of the initial lesion, these findings provided additional support for a primary cervical origin, although an occult ocular or sinonasal primary could not be formally excluded in the absence of dedicated specialist evaluation. Following multidisciplinary gynecologic oncology evaluation, postoperative systemic immunotherapy with pembrolizumab was initiated at a dose of 200 mg intravenously every three weeks. At the 65-day postoperative follow-up, the patient reported minimal vaginal bleeding. Pelvic examination revealed a suspicious lesion at the vaginal cuff (**Figure 2**), with the differential diagnosis including suture granuloma and local tumor recurrence. The lesion was biopsied for histopathological evaluation.

**Figure 2:**
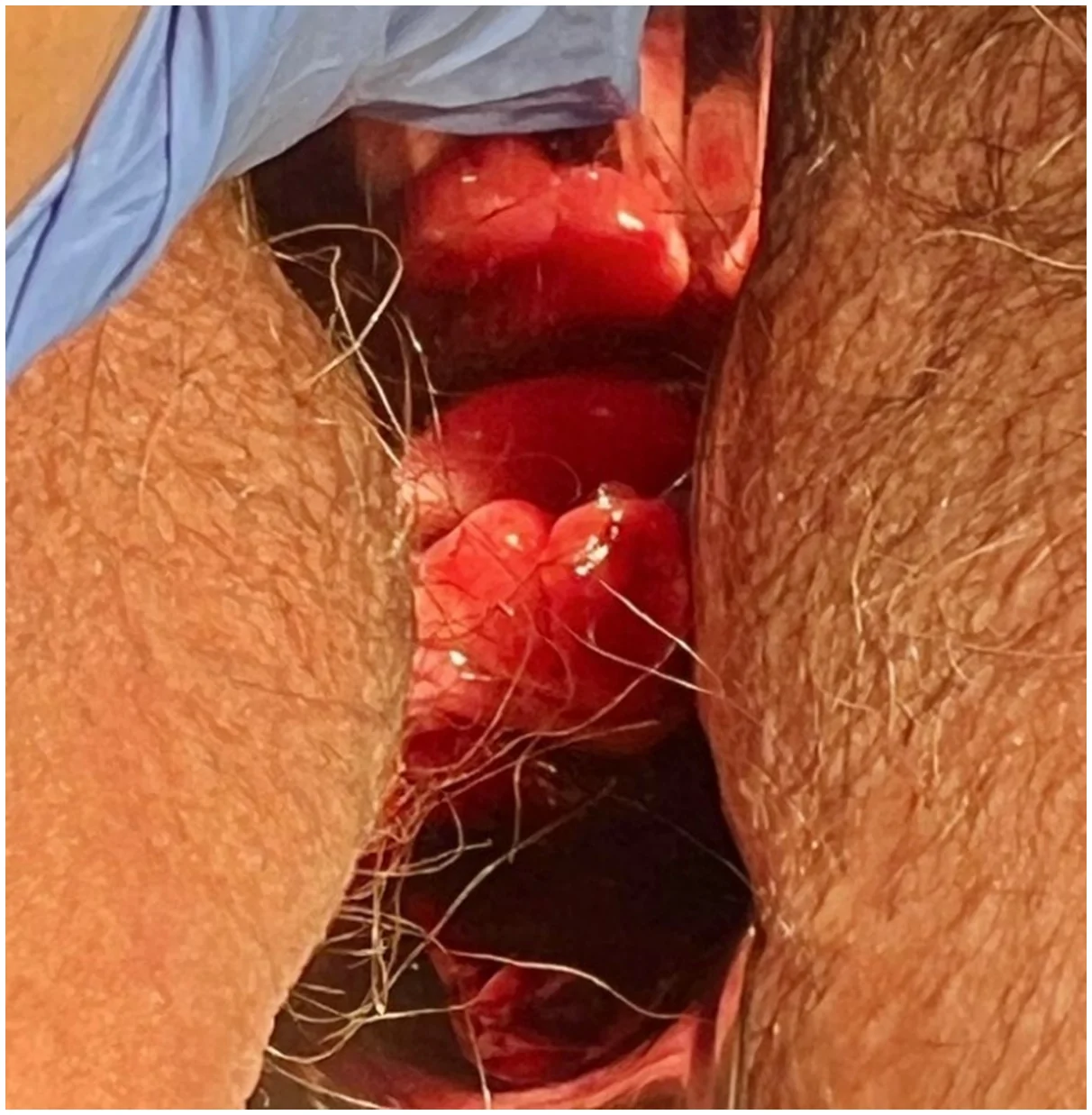
Suspicious lesion at the vaginal cuff identified 65 days after radical surgery and subsequently confirmed histologically as recurrent malignant melanoma.

Histopathological examination of the vaginal lesion confirmed local recurrence of malignant melanoma. Microscopic evaluation demonstrated a diffuse infiltrative proliferation of markedly pleomorphic cells with scant eosinophilic cytoplasm, hyperchromatic nuclei, and frequent mitotic figures, forming confluent sheets within the subepithelial tissue. Immunohistochemical analysis showed diffuse positivity for SOX10 and PRAME, whereas AE1/AE3 was negative in the tumor cells, with preserved staining in the overlying squamous epithelium. In the context of the previous cervical melanoma, these findings confirmed local recurrence. Representative hematoxylin and eosin-stained sections of the recurrent vaginal melanoma are shown in **Figure 3**.

**Figure 3:**
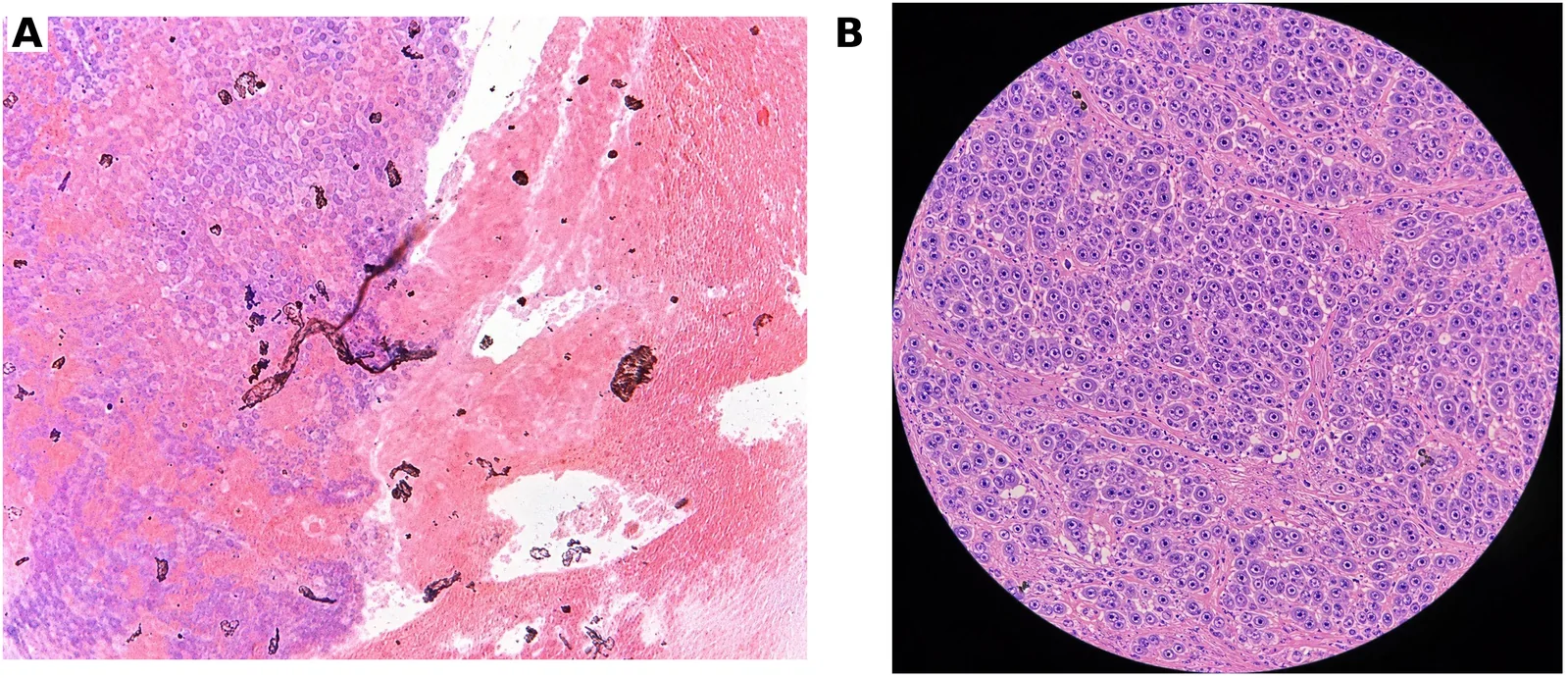
Histopathological features of the recurrent vaginal malignant melanoma. **(A)** Hematoxylin and eosin-stained section showing the recurrent tumor at lower magnification. **(B)** Higher-magnification view demonstrating the cellular morphology of the malignant proliferation.

The biopsy-confirmed vaginal recurrence prompted further evaluation of disease extent. Subsequent ^18^F-FDG PET/CT demonstrated metabolically active confluent lesions involving the vaginal cuff and vagina. A metabolically active left external iliac nodal mass was also identified and considered highly suspicious for secondary involvement. No metabolically active supradiaphragmatic lymphadenopathy, pulmonary lesions, peritoneal nodules, ascites, or metabolically active osseous lesions were identified. Several of the previously documented hepatic lesions, which had remained radiologically stable for two years, subsequently demonstrated increased metabolic activity, raising suspicion of metastatic involvement; however, in the absence of histological confirmation, their nature remained uncertain. Dedicated hepatic MRI and histological confirmation were not available.

Following multidisciplinary oncological evaluation, palliative external-beam radiotherapy was recommended for hemostatic and locoregional control. The patient subsequently received volumetric modulated arc therapy (VMAT) targeting the vaginal cuff and vagina, to a total dose of 30 Gy delivered in 10 fractions of 3 Gy, with good treatment tolerance. Pembrolizumab was subsequently continued at 200 mg every three weeks. During further treatment, the patient developed immune-mediated pneumonitis attributed to pembrolizumab, requiring systemic corticosteroid therapy. At the latest documented follow-up, approximately 16 months after radical surgery, the patient was alive and remained under oncological care.

To provide a concise overview of the diagnostic work-up, therapeutic interventions, and subsequent disease evolution, the key events in the patient’s clinical course are summarized chronologically in **Table 2**.

**Table 2:** Timeline of the patient’s clinical course.

Time point	Key findings and management
Initial presentation	Vaginal bleeding; endocervical polyp excised.
Diagnosis	Cervical malignant melanoma; SOX10/PRAME positive, Ki-67 40–45%; BRAF negative.
Initial radiological staging	No radiologically evident metastatic disease; previously known stable hepatic lesions.
Radical surgery	Radical hysterectomy with bilateral pelvic and para-aortic lymphadenectomy and omental biopsy; bilateral external iliac nodes included; no residual melanoma or nodal involvement.
Postoperative day 2	Dermatological assessment; three suspicious pigmented lesions biopsied, with no evidence of cutaneous melanoma.
Postoperative treatment	Pembrolizumab 200 mg IV every three weeks initiated.
Postoperative day 65	Vaginal bleeding and vaginal cuff lesion; biopsy confirmed recurrent malignant melanoma.
Restaging	PET/CT demonstrated vaginal/cuff disease, suspected left external iliac nodal involvement, and metabolically active hepatic lesions of uncertain nature.
Subsequent treatment	Hemostatic and locoregional VMAT, 30 Gy/10 fractions; pembrolizumab subsequently continued.
Later follow-up	Immune-mediated pneumonitis attributed to pembrolizumab, requiring systemic corticosteroid therapy.
Latest follow-up (~16 months after surgery)	Patient alive and under oncological care.

## Discussion

Primary malignant melanoma of the uterine cervix (PMMC) remains one of the rarest malignancies of the female genital tract. The most comprehensive systematic review published to date by Kechagias et al. identified 137 patients across 96 reports, with a median age of 58 years and a predominance of postmenopausal women. Vaginal bleeding was the most common presenting symptom, reported in approximately 83% of patients (1). Our patient, a 67-year-old postmenopausal woman presenting with vaginal bleeding, therefore reflects the typical demographic and clinical presentation described in the literature. However, the initial appearance of the tumor as an endocervical polyp illustrates how deceptively benign the first clinical manifestation of this aggressive malignancy may be.

The possibility of a primary cervical melanoma was historically questioned because melanocytes were initially considered absent from the cervical epithelium. Subsequent histological identification of melanocytes within the cervix provided a biological basis for melanoma arising at this site (1, 2). Classical criteria proposed to support a primary cervical origin include the presence of melanin within the cervical epithelium, junctional changes adjacent to the lesion, absence of melanoma elsewhere, and a metastatic pattern compatible with a cervical primary (1, 2). In the present case, dermatological assessment and systemic imaging identified no alternative primary melanoma or evidence of distant metastatic disease at initial staging. Although the available histopathological documentation did not allow definitive assessment of junctional activity or an intraepithelial melanocytic component, the cervical localization of the initial lesion together with the absence of another identifiable primary site strongly favored a primary cervical origin.

Nevertheless, two aspects limit the certainty with which a primary cervical origin can be established. First, although dermatological assessment and systemic imaging did not identify an alternative primary site, dedicated ophthalmologic and otorhinolaryngologic evaluations for occult ocular or sinonasal mucosal melanoma were not documented. Second, the hepatic lesions warrant particular consideration. Although they had been present and radiologically stable for approximately two years before the diagnosis of cervical melanoma, several subsequently demonstrated metabolic activity on PET/CT. Dedicated hepatic MRI and histological confirmation were not available; therefore, metastatic involvement could not be definitively excluded. Their previous radiological stability argues against, but does not eliminate, this possibility. Taken together, the available findings favor a primary cervical origin while acknowledging these diagnostic limitations.

Histopathological diagnosis may be particularly difficult in poorly differentiated or amelanotic lesions. Yin et al., in their clinicopathological review of primary cervical melanoma, emphasized the importance of immunohistochemistry in distinguishing these tumors from poorly differentiated carcinoma, sarcoma, and other high-grade cervical malignancies (8). In our case, the combination of diffuse SOX10 and melanoma-cocktail positivity, strong PRAME expression, and absence of epithelial, neuroendocrine, lymphoid, Müllerian, and squamous markers provided strong evidence of melanocytic differentiation. The high Ki-67 proliferation index of approximately 40–45% was additionally consistent with a highly proliferative tumor phenotype. Importantly, the subsequent vaginal recurrence reproduced the melanocytic immunophenotype, with diffuse SOX10 and PRAME positivity and absence of AE1/AE3 expression, providing histological confirmation of recurrent disease.

Mucosal melanoma differs biologically and molecularly from its cutaneous counterpart. In particular, BRAF alterations, which represent an established therapeutic target in cutaneous melanoma, are less frequently encountered in mucosal disease, whereas alterations involving KIT, NRAS, and other signaling pathways have been described (3). No detectable BRAF mutation was identified in our patient, precluding BRAF-directed targeted therapy. This finding further illustrates the therapeutic limitations encountered in mucosal melanoma and supports the potential value of broader molecular profiling, particularly in patients who develop early progression despite systemic treatment.

Despite the fact that a substantial proportion of patients are diagnosed with apparently localized disease, PMMC is characterized by an unfavorable clinical course. Kechagias et al. reported that most patients in their systematic review were diagnosed at FIGO stages I or II, yet mortality remained substantial and recurrent disease was frequent (1). Similarly, Min et al. analyzed 149 published cases and found that radical hysterectomy-based surgery, absence of lymph-node metastases, and lymphadenectomy were associated with improved survival (2). These findings support surgery as the principal treatment for resectable disease, although the retrospective nature and heterogeneity of the available evidence preclude definitive recommendations regarding the optimal extent of surgery. Our patient underwent radical hysterectomy with bilateral pelvic and para-aortic lymphadenectomy in the setting of apparently localized disease, including bilateral external iliac nodal dissection, with no malignant involvement of the examined lymph nodes and no residual melanoma identified in the surgical specimens. Nevertheless, biopsy-confirmed vaginal recurrence developed within a short postoperative interval, emphasizing that apparently complete local treatment does not eliminate the risk of rapid disease progression.

The optimal role of immune checkpoint inhibition in PMMC remains particularly uncertain. Evidence is largely restricted to individual case reports and extrapolation from mucosal melanoma arising at other anatomical sites. In a pooled analysis of mucosal melanoma, D’Angelo et al. reported an objective response rate of 23.3% with nivolumab monotherapy, substantially lower than the 40.9% observed in cutaneous melanoma, illustrating the comparatively limited sensitivity of mucosal melanoma to PD-1 blockade (4). Although these data cannot be directly extrapolated to cervical melanoma, they provide important biological context for the variable responses observed in this exceptionally rare disease.

Published experiences with pembrolizumab in PMMC illustrate this heterogeneity particularly well. Kim et al. reported a patient treated with radical surgery followed by pembrolizumab who developed pulmonary and bone metastases and died eight months after diagnosis, suggesting limited benefit from postoperative PD-1 inhibition in that case (5). In contrast, Zhang et al. reported in 2024 a patient with stage IIB PMMC treated with multimodal therapy followed by 18 cycles of pembrolizumab who achieved complete clinical remission and remained recurrence-free at 31 months (6). More recently, Yu described in 2026 an early-stage, node-negative cervical melanoma that progressed rapidly despite radical surgery and adjuvant anti-PD-1 therapy, ultimately resulting in death 17 months after diagnosis (7). Together, these contrasting reports illustrate the marked variability in response to immune checkpoint blockade and the current inability to reliably identify which patients with PMMC are most likely to derive durable benefit.

Our case adds to this emerging evidence by documenting an early, histologically confirmed vaginal recurrence following radical surgery and postoperative pembrolizumab. Subsequent PET/CT demonstrated metabolically active disease involving the vaginal cuff and vagina, together with a highly suspicious external iliac nodal mass and metabolically active hepatic lesions. Although the nodal and hepatic findings were not histologically confirmed and should therefore be interpreted as suspected metastatic involvement, the short interval between apparently localized disease and the development of multifocal abnormalities indicates an aggressive clinical course. The very short interval between surgery, initiation of postoperative pembrolizumab, and histologically confirmed recurrence should not necessarily be interpreted as failure of PD-1 blockade. Rather, the early appearance of recurrent and radiologically suspected metastatic disease may indicate the presence of clinically occult micrometastatic disease at the time of surgery. The exact interval between the first pembrolizumab administration and recurrence, as well as the number of cycles administered before recurrence, could not be reliably established from the available records, limiting further interpretation of treatment response. The early recurrence observed despite pembrolizumab is particularly relevant when considered alongside the contrasting outcomes reported by Kim et al., Zhang et al., and Yu, reinforcing the heterogeneity of response to PD-1 blockade in cervical mucosal melanoma (5–7).

Radiotherapy remains primarily a strategy for locoregional control or palliation in recurrent or unresectable disease, as evidence supporting a survival benefit in PMMC remains limited (1, 2). In our patient, the development of symptomatic locoregional recurrence led to external-beam radiotherapy with palliative and hemostatic intent. VMAT directed to the vaginal cuff and vagina was well tolerated and provided a rational approach to local symptom control within a sequential multimodal treatment strategy. The course of this patient therefore illustrates the complexity of PMMC management, in which surgery, systemic immunotherapy, and radiotherapy may all be required within a relatively short period because of rapid disease evolution. Pembrolizumab was continued following radiotherapy; however, subsequent development of immune-mediated pneumonitis requiring systemic corticosteroid therapy further illustrates the need to balance potential therapeutic benefit against clinically relevant immune-related toxicity during prolonged checkpoint inhibition.

This report has several limitations. The suspected nodal and hepatic metastatic lesions detected on PET/CT were not histologically confirmed, and dedicated hepatic MRI was not available. Furthermore, dedicated ophthalmologic and otorhinolaryngologic evaluations for occult ocular or sinonasal primary melanoma were not documented. The available histopathological documentation did not permit assessment of several conventional pathological parameters, including precise tumor thickness, depth of stromal invasion, ulceration status, lymphovascular invasion, and a standardized mitotic rate, nor did it allow definitive assessment of junctional activity or an intraepithelial melanocytic component. Additional molecular profiling beyond BRAF, including NRAS and KIT, and PD-L1 assessment were not available. Finally, the exact timing and cumulative number of pembrolizumab cycles administered before recurrence could not be reliably established. Despite these limitations, the cervical localization and melanocytic immunophenotype of the initial tumor, negative dermatological assessment, absence of another primary site on systemic imaging, and subsequent histologically confirmed vaginal recurrence collectively favor a primary cervical melanoma.

## Conclusions

Primary malignant melanoma of the uterine cervix remains a diagnostic and therapeutic challenge. This case illustrates its aggressive clinical behavior, with early biopsy-confirmed recurrence and rapid progression following surgical treatment and immunotherapy. The unpredictable course of this rare malignancy underscores the importance of close surveillance and multidisciplinary management, while highlighting the need for further evidence to guide therapeutic decisions.

## Patient perspective

The patient described the diagnosis as unexpected and initially difficult to understand. Despite the complexity of the disease and its treatment, she reported an overall improvement in her condition following the most recent therapy.

## Data Availability

The original contributions presented in the study are included in the article/supplementary material. Further inquiries can be directed to the corresponding author.
